# CHA2DS2-VASc Calculations in Patients with Atrial Fibrillation receiving oral anticoagulants in Scotland: an analysis of linked administrative data

**DOI:** 10.23889/ijpds.v1i1.287

**Published:** 2017-04-19

**Authors:** Wenjie Zeng, Tanja Mueller, Brian Godman, Samantha Alvarez-Madrazo, Marion Bennie

**Affiliations:** School of Economics and Management, Chongqing Jiaotong University; University of Strathclyde

## Objectives

Patients with atrial fibrillation (AF) have an increased risk of developing stroke, and oral anticoagulants (OACs) are commonly used in stroke prevention. The CHA2DS2-VASc score has been proved to be a simple and effective tool for stroke risk assessment which guides the selection of treatment with OACs. This score is calculated using disease diagnoses; however, a range of different versions exist, making comparability questionable.

The aim of this study was to compare CHA2DS2-VASc scores in OAC treated patients with a hospital-confirmed diagnosis of AF in Scotland using different subsets of ICD-10 codes.

## Approach

This is a retrospective study, covering AF patients in Scotland who received at least one prescription for any OAC between January 2009 and June 2014. The Prescribing Information System (PIS) was used to identify patients with OAC prescriptions, while the Scottish Morbidity Records (SMR) provided patients’ diagnoses. Different sets of ICD-10 codes, using varying definitions, were used to classify the stroke risk in AF patients in order to account for heterogenous definitions applied in previously published studies. The main differences in codes used were the inclusion or exclusion of unclassified stroke (ICD-10 code I64), and the inclusion of pulmonary embolism (PE) (I26) as either ``prior thromboembolic event'' or ``vascular disease''.

## Results

In our study, a cohort of 71012 AF patients with OAC prescription were analysed. Using narrow disease definitions, 18.15% of patients were categorised as being at low risk of stroke (score 0-1), 74.80% at medium risk (score 2-5), and 7.04% at high risk (score 6-9). With an extended disease definition, including PE in ``prior thromboembolic event'', 14.81% of patients were at low risk (score 0-1), 72.60% at medium risk (score 2-5), and 12.59% at high risk (score 6-9); while including PE in ``vascular disease'', 14.99% of the patients had a score 0-1, 73.54% a score 2-5, and 11.48% a score 6-9.

## Conclusion

The change in score definitions makes a difference mainly in the number of patients categorised as very low risk or high risk. Standardisation in ICD-10 code definition of risk diseases could be useful in order to make the results comparable from different studies and further evaluate OAC choice according to risk group.

